# Exploring resource scarcity and contextual influences on wellbeing among young refugees in Bidi Bidi refugee settlement, Uganda: findings from a qualitative study

**DOI:** 10.1186/s13031-020-00336-3

**Published:** 2021-01-07

**Authors:** Carmen H. Logie, Moses Okumu, Maya Latif, Daniel Kibuuka Musoke, Simon Odong Lukone, Simon Mwima, Peter Kyambadde

**Affiliations:** 1 Factor Inwentash Faculty of Social Work, University of Toronto, 246 Bloor Street West, Toronto, ON M5S 1V4 Canada; 2 Women’s College Research Institute, Women’s College Hospital, 76 Grenville St, Toronto, ON M5G 1N8 Canada; 3 School of Social Work, University of North Carolina, Chapel Hill, 325 Pittsboro St, Chapel Hill, NC 27599-3550 USA; 4 International Research Consortium, Kampala, Uganda; 5 Uganda Refugee & Disaster Management Council, Yumbe, Uganda; 6 AIDS Control Program, Ministry of Health, Plot 6, Lourdel Road, Nakasero, Kampala, Uganda; 7 Most At Risk Population Initiative Clinic, Mulago Hospital, Kampala, Uganda

**Keywords:** Uganda, Refugee youth, Adolescents, COVID-19, Sexual and gender-based violence, Substance use, Water insecurity, Food insecurity, Psychosocial stress

## Abstract

**Background:**

Contextual factors including poverty and inequitable gender norms harm refugee adolescent and youths’ wellbeing. Our study focused on Bidi Bidi refugee settlement that hosts more than 230,000 of Uganda’s 1.4 million refugees. We explored contextual factors associated with wellbeing among refugee adolescents and youth aged 16–24 in Bidi Bidi refugee settlement.

**Methods:**

We conducted 6 focus groups (*n* = 3: women, *n* = 3: men) and 10 individual interviews with young refugees aged 16–24 living in Bidi Bidi. We used physical distancing practices in a private outdoor space. Focus groups and individual interviews explored socio-environmental factors associated with refugee youth wellbeing. Focus groups were digitally recorded, transcribed verbatim, and coded by two investigators using thematic analysis. Analysis was informed by a social contextual theoretical approach that considers the interplay between material (resource access), symbolic (cultural norms and values), and relational (social relationships) contextual factors that can enable or constrain health promotion.

**Results:**

Participants included 58 youth (29 men; 29 women), mean age was 20.9 (range 16–24). Most participants (82.8%, *n* = 48) were from South Sudan and the remaining from the Democratic Republic of Congo (17.2% [*n* = 10]). Participant narratives revealed the complex interrelationships between material, symbolic and relational contexts that shaped wellbeing. Resource constraints of poverty, food insecurity, and unemployment (material contexts) produced stress and increased sexual and gender-based violence (SGBV) targeting adolescent girls and women. These economic insecurities exacerbated inequitable gender norms (symbolic contexts) to increase early marriage and transactional sex (relational context) among adolescent girls and young women. Gendered tasks such as collecting water and firewood also increased SGBV exposure among girls and young women, and this was exacerbated by deforestation. Participants reported negative community impacts (relational context) of COVID-19 that were associated with fear and panic, alongside increased social isolation due to business, school and church closures.

**Conclusions:**

Resource scarcity produced pervasive stressors among refugee adolescents and youth. Findings signal the importance of gender transformative approaches to SGBV prevention that integrate attention to resource scarcity. These may be particularly relevant in the COVID-19 pandemic. Findings signal the importance of developing health enabling social contexts with and for refugee adolescents and youth.

## Background

Forty percent of the world’s 79.9 million refugees are under 18 years old. Refugee and displaced persons disproportionately experience poverty, overcrowded living conditions, and poor sanitation that elevate food and water insecurity challenges [1, 2]. These challenges are heightened during pandemics such as COVID-19 [3, 4]. Resource scarcity conceptualizes shared social, economic, and ecologic vulnerabilities to water and food insecurity, coping strategies, and impacts on wellbeing [5]. How well resource scarcity frameworks reflect refugee youth experiences is underexplored. Uganda is an important context to understand experiences of research scarcity and linkages to wellbeing among refugee and displaced adolescents and youth as sub-Saharan Africa’s largest refugee hosting country with more than 1.4 million refugees [6].

Food and water insecurity are among the biggest threats to human survival and wellbeing [4, 7]. Wutich and Brewer conceptualized the resource scarcity theoretical framework that bridges understanding of vulnerabilities to, and impacts of, food and water insecurity [5]. Drivers of food and water insecurity include ecological factors such as seasonal changes, climate changes, drought, and flooding [5, 8]—communities navigate these stressors differently. This signals that ecological approaches alone cannot explain community vulnerability or resilience to resource insecurity. Socio-economic factors, including poverty, access to education, alongside social factors such as inequitable gender norms, also drive experiences of food and water insecurity [5, 8]. Stress from uncertainty and unpredictability about acquiring sufficient food and/or water can contribute to anxiety and depression [5, 8]. For instance, a Ugandan study found that food insecurity was associated with depression [9]. Another Ugandan study reported geospatial clustering of water and depression among women [10]. Limited studies assess resource insecurity and wellbeing among refugees, and specifically among refugee adolescents and youth: a cross-sectional study revealed that lack of food or water was associated with depression among internally displaced adults in Uganda [11].

Resource scarcity is gendered. As women largely engage in household activities that require water—such as cooking, cleaning, and clothes washing—they are more likely to be blamed and shamed than men for the inability to meet social cleanliness standards for homes, children and families [12]. Both food and water insecurity have been linked with sexual and gender-based violence (SGBV). Poverty has been linked with increased SGBV in post-conflict Sierra Leone [13], Liberia [13], Colombia [14], and post-earthquake Haiti [15]. Urban refugee adolescent girls and young women had 7-fold higher odds of experiencing violence when food insecure in Kampala, Uganda [16]. Hatcher’s conceptual framework links food insecurity to SGBV via physiological, psychological, relational, and social pathways [17]. Women also report sexual assault concerns on journeys to collect water [18–22]. COVID-19 physical distancing restrictions may result in collecting water alone [23], further elevating risks of violence exposure. There are knowledge gaps at large regarding adolescents’ experiences of resource scarcity and wellbeing [24]. It is urgent to address this gap: age and gender-related social roles shape experiences of water access and expectations to complete water-related tasks. Unaddressed stressors in adolescence, including poverty, conflict and violence, have life-long harmful impacts on wellbeing [25, 26]. Researchers call attention to the importance of understanding contextual drivers of SGBV among adolescent girls and young women in low and middle income countries (LMIC) [27], and underscore the need for further research on SGBV prevention needs for adolescent girls and young women in humanitarian settings [28].

COVID-19 poses significant risks for wellbeing among refugee and displaced persons [29, 30]. Poverty, overcrowded living conditions, and poor sanitation elevate forcibly displaced persons’ COVID-19 risks while limiting the ability to practice mitigation strategies such as handwashing and physical distancing [30–32]. COVID-19’s psychosocial impacts may exacerbate existing stress [33]. This is particularly salient to explore with refugees who may experience a high prevalence of psychological distress [34]. There are rising concerns of increased SGBV during COVID-19, including in humanitarian contexts [35, 36]. Little is known of COVID-19-related stressors among refugee and displaced adolescents and youth, and how these may amplify pre-existing stressors.

To address these knowledge gaps regarding contextual factors that shape wellbeing and SGBV risks among refugee adolescents and youth, we conducted a qualitative study in Bidi Bidi refugee settlement in Uganda [37]. Specifically, our study explored refugee adolescent and youth perspectives on SGBV vulnerabilities and drivers in Bidi Bidi. Our secondary aim, due to emergence of COVID-19 during our study, was to explore experiences and perspectives toward COVID-19 among this population.

## Methods

### Participants

This qualitative study was a collaboration between academics, Uganda Refugee & Disaster Management Council (URDMC), and the Ugandan Ministry of Health to explore young refugees’ wellbeing in Bidi Bidi settlement, Yumbe District, Uganda. Inclusion criteria were: identifying as a refugee or displaced person aged 16–24; living in Bidi Bidi settlement (Zone 3); fluent in speaking English, Luganda or Juba Arabic; able to provide informed consent.

### Study setting

Bidi Bidi is the 2nd largest refugee settlement in the world, with 232,743 residents in 42,754 households; 22% (*n* = 51,072) of residents are youth aged 15–24 and women comprise 53% (*n* = 122,331) of residents [38]. Three-quarters (75.6%) of Bidi Bidi residents are unemployed [38], and 74% of children eligible for primary school were enrolled [39]. Most (59%) heads of households as of October 2020 reported poor or borderline food consumption, suggesting widespread food insecurity [39].

### Recruitment

We worked closely with collaborators at URDMC to hire 12 peer research assistants who we trained in research methods and ethical processes. Peer research assistants identified as refugee young people aged 18–24 living in Bidi Bidi, and they conducted convenience sampling using word of mouth with peers living in Zone 3 to recruit study participants. Study collaborators at URDMC provide youth friendly information and programs, and peer research assistants shared the study information with their own social networks as well as participants in these programs. This study focused on Zone 3 as it has the largest population (*n* = 55,333) of Bidi Bidi’s five zones [38]. The study was approved by the Research Ethics Boards at the University of Toronto, Mildmay Uganda (#REC REF-0221-2019) and Uganda National Council for Science and Technology (#SS-5273). Participants received an honorarium of 40,000 Ugandan shillings (~$10 USD).

### Data collection procedures

This qualitative study involved 6 focus groups (3 with young women, 3 with young men) and ten in-depth individual interviews in February 2020 conducted in an outdoor private space using physical distancing. Each focus group was 45–60 min. Focus groups were co-facilitated by a trained qualitative researcher fluent in English and Luganda and a translator fluent in English and Juba Arabic. Our study was designed to understand contextual factors associated with SGBV and youth wellbeing at large. Questions included: *“What is the situation of sexual and gender-based violence like in Bidi Bidi settlement?”*, *“Can you give examples of what sexual and gender-based violence might look like in your community?”*, *“What are community attitudes toward sexual and gender-based violence?”*, *“What are some of the causes behind sexual and gender-based violence in your community?”*, and *“Who is most vulnerable to sexual and gender-based violence in your community?”* As COVID-19 became a concern near the end of data collection, after the individual interviews were completed but before the focus groups, we were able to add two COVID-19 questions in the focus groups: *“What do you know about COVID-19?”* and *“How has COVID-19 impacted your community?”*

### Data analysis

Focus groups were conducted in English and Juba Arabic, digitally recorded, transcribed verbatim, and translated into English. Data were coded by two investigators using thematic analysis [40, 41]. Thematic analysis is a theoretically flexible approach that involves several readings of transcripts to note preliminary ideas, followed by producing initial codes, and then collating codes into themes [41].

Analysis was informed by Campbell and Cornish’s [42] conceptualization of the social context and its role in shaping health promoting environments. They conceptualized receptive social environments as those in which persons who are marginalized could engage in transformative communication to realize control over their health and wellbeing [42]. Transformative communication is rooted in a critical awareness of social, political and economic factors that produce vulnerability to poor health and wellbeing and can prepare persons to formulate strategies to challenge these inequities [43]. Social contexts shape the ways that people are supported and enabled—or unsupported and constrained—to engage in health promoting practices [15, 44]. Interrelated dimensions of social context that facilitate or inhibit transformative communication include symbolic, material and relational contexts [42]. Symbolic dimensions of context include sociocultural worldviews, meanings, and beliefs that shape understanding of oneself and others, including who is recognized as valuable and worthy of dignity and respect in society [42, 45]. This includes gender ideologies and the ability of all genders to realize their potential and take agency to make decisions over their lives. Material contexts are closely linked with symbolic contexts and include resources that influence agency, for instance, access to employment, food, and other survival necessities [42]. These material resources are not only health promoting but also increase symbolic dimensions of recognition of value, dignity, and worth. Material contexts also include the opportunities and ability to practice skills and agency. Finally, relational contexts shape the ability for people to realize their rights, participate as leaders in their community, and achieve financial independence [42]. Social networks and social participation opportunities are also dimensions of relational contexts that can foster empowerment and agency to be active participants in social arenas [42].

## Results

The 48 participants included 58 youth (29 men; 29 women), the mean age was 20.9 (range 16–24). Most participants (82.8%; *n* = 48) were from South Sudan and the remaining from the Democratic Republic of Congo (17.2% [*n* = 10]). Further details about participants are provided in Table 1.

**Table 1:** Overview of socio-demographic characteristics among refugee adolescent and youth participants in Bidi Bidi refugee settlement, Uganda (*n* = 58)

	Focus group participants (*n* = 48)	In-depth interview participants (*n* = 10)
**Age** (mean, standard deviation)	20.9 (1.8)	20.8 (2.7)
**Duration of Years in Uganda**		
Mean (range)	2.05 (0.5–4.0)	2.5 (1–4.0)
	*N* (%)	*N* (%)
**Gender**		
Men	24 (50.0)	5 (50.0)
Women	24 (50.0)	5 (50.0)
**Country of Birth**		
South Sudan	41 (85.4)	7 (70.0)
Democratic Republic of Congo	7 (14.6)	3 (30.0)
**Level of Education**		
Less than Primary	15 (31.2)	3 (30.0)
Less than Secondary	21 (43.7)	6 (60.0)
Completed Secondary	8 (16.7)	–
Attended Technical College	2 (4.2)	–
Some University	2 (4.2)	–
University Degree	–	1 (10.0)
**Current Employment Status**		
Unemployed	37 (77.1)	10 (100.0)
Employed	3 (6.3)	–
Self-employed	8 (16.7)	–

Participant narratives revealed the complex interrelationships between material, symbolic, and relational contexts that shaped wellbeing via SGBV exposure. Water, food scarcity, environmental degradation, and unemployment (material contexts) produced stress. These economic insecurities exacerbated inequitable gender norms (symbolic contexts) to elevate risks for SGBV in community settings and intimate partnerships (relational context). Participants also reported negative community impacts (relational context) of COVID-19 that were associated with fear and panic alongside increased social isolation due to business, school, and church closures.

### Material contexts and resource scarcity

Upon settling in Bidi Bidi, living conditions for many residents were shaped by pervasive economic precarity and concomitant resource scarcity. Familial discord was perceived to be instigated by conditions of poverty, beginning in experiences of conflict and war and continuing into resettlement as refugees. As a young woman explained,

“The war has stripped many of their privileges in their home country so normally poverty makes them fight in homes. Lack of food at homes is another source of fighting. When the supply is not enough, a lot of disagreements crop up that lead people into fights at home. This is worsened by the polygamous nature of marriages that is common in our communities.” (Young Woman, Interview A)

Several participants described stressors caused by poverty that included escalated risks of intimate partner violence. For instance, a young woman explained: “poverty is also another source of violence; for instance, lack of food at home for children can easily ignite a fight between the wife and husband.” (Young Woman, Interview B) When husbands were not able to provide material resources like money or food, this caused tensions that increased intimate partner violence: “One of the causes of sexual and gender-based violence is poverty. For instance, when the woman asks for money from the husband and if the man says he does not [have] this can also lead to sexual and gender-based violence.” (Young Man, Interview C) This view was substantiated by another young woman, who noted that “poverty causes a lot of domestic violence in homes, a woman can ask the man for money and when the man is broke or poor [with] no money this can make him lose his temper hence causing fights” (Young Women Focus Group [FG] 1).

A dearth of consistent, meaningful employment, vocational, and recreational activities — leading to what participants termed “idleness” — was linked to SGBV. As articulated by one young man, “poverty pushes people to fight because they are angry about their situations and idleness” (Young Men FG 3). This understanding was reinforced by another young man, who reported “idleness makes people to commit such crimes because they basically have nothing to keep them busy and occupied. Hence, they resort to such behaviors” (Young Man, Interview D). Again connecting violence to experiences of loss and a lack of meaningful work, a young woman explained, “people are unemployed and idle so they end up doing evil in frustration. Back home, many of these people owned property but now, here they are helpless and feel frustrated and are desperate” (Young Women FG 3). When asked for solutions to curbing SGBV, a young woman described that “there should be training, entertainment and community engagement in different activities to reduce idleness that sometimes fuels violence.” (Young Woman, Interview E) While unemployment was understood as a driver of SGBV, unequal power dynamics between host (Ugandan national) and refugee community members could also contribute to community violence. As one young man described during harvest season: “the nationals are not friendly in times of cultivation, so when you go to the farm, you are in danger of being raped or abused” (Young Men FG 3). Thus, in addition to unemployment, working conditions may also contribute to SGBV risks for refugee youth in Bidi Bidi.

### Relational contexts and resource scarcity

Gender norms concerning resource acquisition and related expectations surrounding sex also shaped relationship dynamics. A lack of household resources was commonly invoked to account for why adolescent girls and young women engaged in transactional sex. When asked about the causes of SGBV in the community, one man elaborated that “poverty and lack of enough food. When it reaches night and a woman refuses sex it can lead to beating and being raped. Lack of food at home can cause a woman to go out for prostitution in the night in order to get food for her family” (Young Men FG 2). Another woman noted how the inability to purchase basic supplies meant that “girls are forced out to find their own ways of survival, and to get things like pads, lotions, etc. and this makes them vulnerable to men who end up luring them into sex” (Young Women FG 1). Across participant narratives, transactional sex was articulated as a practice that was exclusively engaged in by women.

A lack of economic resources was seen as contributing to early marriage to ease financial strains felt by families. As a young woman explained, “poverty is one of the causes of SGBV in our community. Most people here force their young girls to get married at early age against their will just to get rich and this is very common in the settlement” (Young Women FG 2). Another participant reiterated poverty’s negative impacts on adolescent girls and young women: “There is poverty where resources are not enough, bringing desperate measures taken by young girls like prostitution, early marriages, and even spouses fighting.” (Young Men FG 3) Early marriage was also described as a phenomenon that occurred at the nexus of poverty and sexual violence: “sometimes parents cause some of the abuses such as early marriages by pushing their girls to get married to the people who rape them. This is probably due to sometimes culture and other times poverty of the parents” (Young Woman, Interview D).

Although early marriage was mostly described as affecting young women, young men also described feeling pressure to marry at early ages. As one participant explained, “boys and girls are forced to marry. They [are] even rebuked when compared to their peers who have already married at a certain age … normally between 15-16 years for girls and 18 years for boys” (Young Man, Interview F). However, while early marriages affected both young women and men, participant narratives reflected gender differences in underlying motivations. For young men, early marriages were described as being related to normative understandings of the progression of a life course, specifically that one ought to be married by a certain age. With young women, however, early marriage occurred largely as mechanism of resource acquisition for her family. As a young woman articulated: “girls are seen as source of wealth, thus many are forced to marry” (Young Women FG 1). As described above, early marriages of girls may also occur in response to an instance of sexual assault, demonstrating the long-lasting effects such violence can have.

### Symbolic contexts and resource scarcity

Inequitable gender norms and practices such as dowry underpinned SGBV targeting adolescent girls and young women. As one youth elaborated:

Our culture elevates men more than women, thus many rules are against women and girls. All these are factors that lead to SBGV in our communities. For example, a man cannot for any reason be denied sex when he paid dowry. This can cause a serious fight, even when it comes after assault for something else, and can cause reporting to elders. In most cases when it comes to sex related matters in wedded families the women are on the losing end. (Young Man, Interview F)

Reporting instances of such SGBV may not necessarily ameliorate situations or result in accountability. As a young woman described, “In instances of marital rape, when wives report such cases relatives support the man instead and the lady is usually punished by making her pay a goat for denying herself to her husband” (Young Women FG 1). As another participant similarly articulated: “In my village if a man asks a woman for sex and she refuses the woman will be reported to the clan members and pays either a goat or money. The men say they have owned the women so they can demand for whatever they want anytime” (Young Men FG 1). Thus, inequitable gender norms, including the sense of debt, obligation, and ownership that stems from initial dowry payments, can reproduce power imbalances in intimate and familial relationships.

Gender norms also shape the division of labour, whereby subsistence strategies of water and firewood collection are primarily performed by women and girls. As a young man recounted, “the collection of firewood from the bush has exposed women and girls to the risk [of violence]. They are afraid now. There are stories and news we have got about women being chased after and raped here in Bidi Bidi” (Young Men FG 1). The cover provided by dense bush thickets and forest provide an environment that increased risk of sexual assault, especially at night. This was illustrated in an account of sexual assault experienced by a young woman, who recalled: “what I know is especially rape which happened to me. I went to fetch water and a man came from behind and forced me and raped me. He ran away and was never found” (Young Woman, Interview G). These risks collecting firewood were amplified due to environmental degradation in Bidi Bidi. As a young man reported, “people used to gather firewood nearby, that’s by the time we came here in 2016. However, with time, trees reduced and we started moving farther out of the camp. It’s in these far distances that most of the assaults and rapes cases are reported from.” (Young Man, Interview F). Thus, due to deforestation, women and girls are increasingly forced to walk further distances from their communities to perform these tasks, making them more isolated and therefore more vulnerable to violence.

### Relational contexts and COVID-19

The emergence of COVID-19 produced recommendations that influenced social practices (relational context). These changes included staying home and adjusting the manner of social greetings. For instance, participants discussed staying home as necessary: “we should stay at our homes” (Young Women FG 2) and “yesterday there were announcements of how people should wash their hands frequently, stay home, and not to move in crowded places” (Young Men FG 3). To facilitate hand hygiene, persons reported being advised to stop greeting persons with their hands, “People have also been told not to greet each other using hands and not to touch their eyes, noses, and mouth” (Young Women FG 1). Others described being advised to simply stop greeting persons: “We need to avoid greeting” (Young Men FG 1) and “we should not greet people” (Young Women FG 2). Others specifically mentioned social distancing: “they have been advised to wash hands and keep social distance” (Young Women FG 2). Respondents described that those with mobile phones received these informational COVID-19 text messages: “Those with phones have an idea from the messages sent. Yesterday there were announcements of how people should wash their hands frequently, stay home and not to move in crowded places” (Young Men FG 3).

Fear and panic were described as other impacts of COVID-19, partly due to viewing COVID-19 as serious and incurable. For instance, persons described “It is deadly” (Young Men FG 1) and “It is a deadly disease that kills and has no cure for now” (Young Women FG 1). Participants reported “It has brought fear in people” (Young Women FG 2), contributing to feelings of helplessness (“We are in terrible fear and we don’t know how to go about it” [Young Women FG 3]) and panic (“People are panicking” [Young Women FG 1]). Others expressed that “people want to go back to South Sudan” (Young Women FG 1). This desire was largely rooted in the belief that COVID-19 had not yet reached South Sudan: “If possible, people want to go back to South Sudan instead of dying from here. In South Sudan this disease is not there” (Young Women FG 3) and “corona has made our parents think of going back to South Sudan since corona is not there.” (Young Men FG 2). Participants also reported community closures, “Business has been shut down. Schools have been closed. Churches were closed” (Young Women FG 3), of places that were sources of employment and entertainment. As a participant explained: “There are no gatherings, no video halls and churches which is affecting the community because these places normally entertain us to keep us busy” (Young Men FG 3).

## Discussion

**Fig. 1:**
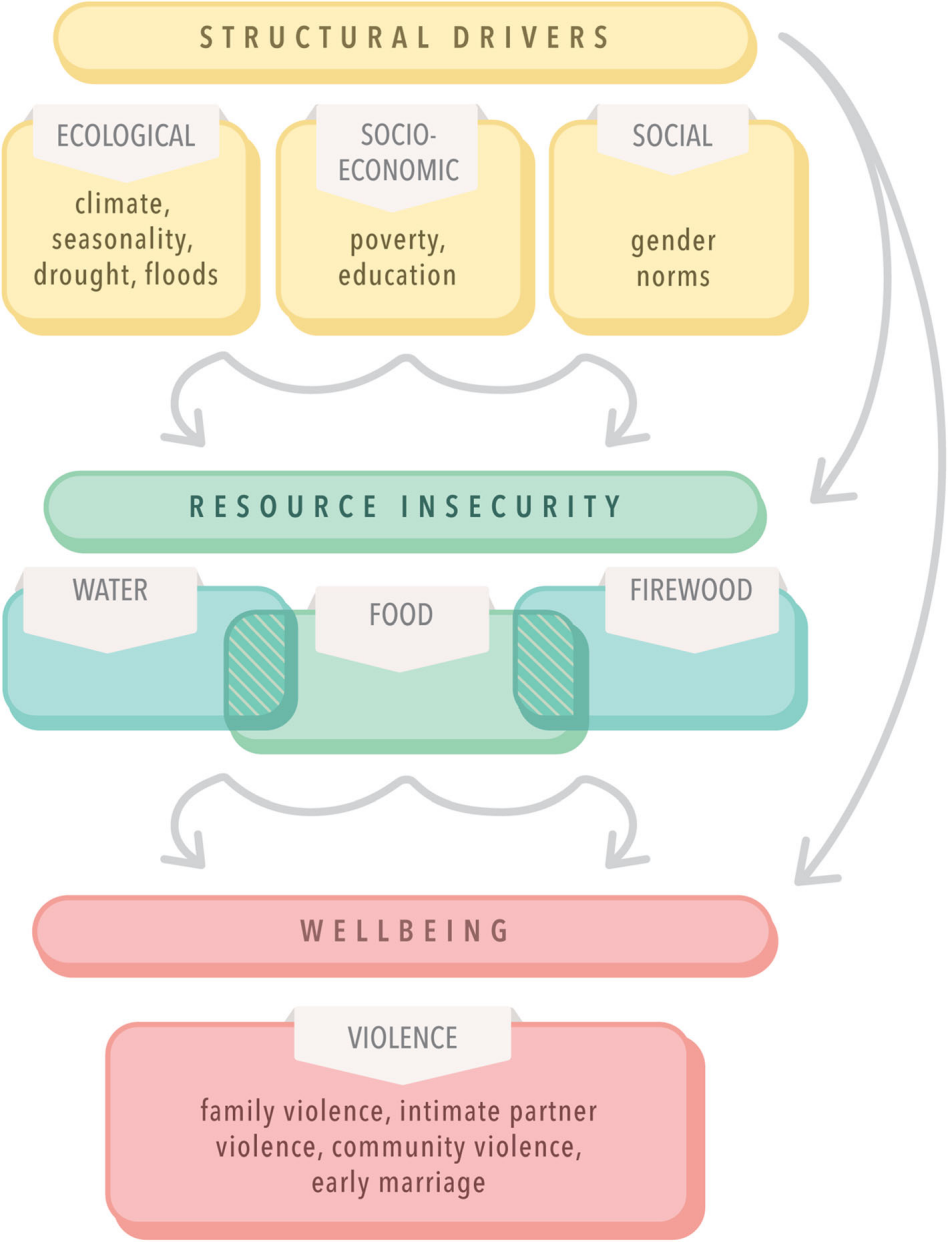
Conceptual model of structural drivers of resource scarcity and wellbeing among refugee adolescents and youth in Bidi Bidi, Uganda

Findings signal the central role of social contexts in shaping the wellbeing and life opportunities of refugee adolescents and youth participants in this study. While initially planned to examine the ways that social contexts shape SGBV vulnerabilities, participant narratives emphasized a range of pathways from environmental stressors to SGBV and wellbeing more broadly. These findings align with the resource scarcity model [5], suggesting its utility for exploring pathways to SGBV among refugee adolescents and youth. Specifically, we found that structural drivers, including ecological (e.g. deforestation), socio-economic (e.g. unemployment), and social (e.g. gender norms) factors produced resource scarcity (water, food, firewood), that in turn produced stressful contexts that contributed to a range of SGBV experiences in families, intimate partnerships, and community contexts. We developed Fig. 1 to reflect the alignment of study findings with the resource scarcity model. By framing the linkages between each of the three contexts (material, relational, symbolic) and resource scarcity with refugee adolescents and youth, our findings suggest the ways that resource scarcities constrain life opportunities in ways that often result in SGBV targeting adolescent girls and women.

In the material context, participant narratives reflected economic marginalization experienced in unemployment that resulted in pervasive and chronic poverty and food insecurity. These resource constraints contributed to household conflict and subsequently increased intimate partner violence, corroborating findings from other studies [8, 17]. The material context also includes the ways that resource constraints inhibit the ability to practice skills and agency and in turn, to experience social recognition [42]. Narratives from refugee adolescent boys and young men reflected frustration at not being able to use skills and participate in society, and being forced into what they termed as “idleness”. This “idleness” not only harmed wellbeing of refugee adolescent girls and women due to its connection with SGBV, but limited refugee adolescent boys and men’s ability to realize their potential and economic agency. Findings reflects prior research with internally displaced youth in Haiti that conceptualizes intersectional violence at the nexus of gender, age, displacement, and poverty [15]. These findings also reflect prior work by Gibbs et al. [45, 46] that describe the ways that poverty and precarious livelihoods among young men can produce hopelessness, powerlessness, and a loss of control due to the inability to access the ideals of youth masculinity.

Inequitable gender norms intersected with material resource scarcity to produce relational contexts in which adolescent girls and young women study participants reported limited agency over their relationships and financial independence. This manifested in our findings in the need for refugee adolescent girls and young women participants to engage in transactional sex to support themselves and/or their families, in addition to forced early marriage by their family due to poverty or their experiences of sexual violence. In such relational contexts, adolescent girls and young women were largely not able to realize their rights, leadership, financial independence, or empowerment [42]. These findings map onto socioecological conceptualizations of SGBV determinants in humanitarian settings that includes relational contexts of men’s control of wealth and increased prevalence of child marriage [47].

Shaping the experiences of material and relational contexts were symbolic contexts where inequitable gender norms reduced the recognition of refugee adolescent girls and young women participants as worthy of value, dignity, and respect. Debt and obligation from dowry payments reduced adolescent girls’ and women’s agency to stop SGBV, including marital rape, or to acquire familial or community support for these situations. This findings align with prior research in a refugee camp in Kenya that described how dowry practices and early marriage constrained agency among women and increased SGBV risks [48]. Gendered divisions of labour resulted in adolescent girls and women largely being responsible for water and firewood collection, in turn exposing them to violence. This was exacerbated by ecological changes such as deforestation. These findings corroborate research on the gendered impacts of resource scarcity due to girls’ and women’s roles collecting water and firewood [12], and risks of SGBV during water fetching [18–22] and firewood collection [49–51]. Our findings suggest the importance of adding firewood to our adapted resource scarcity model (Fig. 1) that originally focused on water and food insecurity [5].

Our findings regarding COVID-19 experiences can be situated in the results above regarding material, relational, and social contexts that shape refugee youth participants’ wellbeing, including experiences of “idleness”, food insecurity, and SGBV. While we collected only a small amount of qualitative data on COVID-19 at the beginning of the pandemic, we learned that COVID-19 was perceived to impact relational contexts in several ways, including changing traditional greetings (no longer hugging and shaking hands), advising people to stay in their homes more, and closing community meeting spaces (such as churches). COVID-19 also contributed to fear and wishes to return to home countries. Together, these findings suggest the importance of further exploring food insecurity and SGBV that may have worsened since COVID-19 across global contexts [3, 4, 35, 36], specifically among refugee adolescents and youth in humanitarian settings to understand their lived experiences. Our findings also suggest the importance of psychosocial support for young refugees who may be experiencing fear, panic and social isolation during COVID-19 that may exacerbate pre-existing psychological distress [33, 34].

Our study has limitations. We conducted non-random sampling and focused on one zone in Bidi Bidi, future studies could include a wider range of participants, including those not connected to refugee support agencies and living in other zones, to gather a wider range of lived experiences. We did not differentiate responses by country of origin, and therefore could not explore differences in lived experiences between participants from South Sudan and from the DRC. While we were able to explore gender differences, we did not have sufficient disaggregated data to identify differences by age or country of origin. We examined a limited number of COVID-19 questions and only in focus groups (not individual interviews), precluding gaining an in-depth understanding of COVID-19 experiences among refugee/displaced youth. Future research could include individual longitudinal interviews to examine how perceptions of COVID-19 have changed over time, and how experiences vary by age, country of origin, and gender. Despite these limitations, our study provides insight into the complexity of social contexts that interact to constrain the opportunities for refugee adolescents and youth in Bidi Bidi to realize wellbeing, including economic security, meaningful livelihoods, and social recognition. For adolescent girls and young women these social contexts increased risks of SGBV and constrained autonomy and agency. Findings also contribute to the nascent evidence base on adolescents’ experiences of resource scarcity and wellbeing in humanitarian settings [24].

## Conclusion

Contextual factors shape wellbeing, agency, and the opportunity to realize health and rights among refugee adolescents and youth participating in our study. The social ecological model for SGBV among adolescent girls in humanitarian settings [47] is a valuable foundation that can be extended to consider resource scarcity (water, food, firewood) and linkages with ecological contexts such as deforestation [5]. Approaches for SGBV reduction with refugee adolescent boys and young men can also consider the interplay between material contexts of resource scarcity and “idleness” with symbolic and relational contexts influenced by traditional gender role expectations [46]. Strategies could address structural drivers of resource scarcity and SGBV alongside developing contextually specific SGBV and health promoting programs [27, 47]. There is a need to build the evidence base of efficacious strategies for reducing SGBV among adolescent girls and young women in humanitarian contexts [28, 47]; while taken as good practice, there is a need to further evaluate system level approaches including referrals, justice and legal aid, safety and risk mitigation (e.g. lighting at water sources) [47]. Other promising approaches with adolescent girls include livelihoods and social asset development, mentorship, and safe spaces; yet these approaches require further rigorous evaluation and study design [28] and gender and age tailored strategies for refugee adolescent boys and young men can also be developed. Together, findings can inform future research and practice focused on developing enabling social environments to promote refugee adolescent and youth wellbeing, including reducing SGBV and resource scarcity, and addressing current and long-term impacts of COVID-19.

## Data Availability

The datasets generated and/or analysed during the current study are not publicly available due to research ethics board restrictions but are available from the corresponding author on reasonable request and on attaining research ethics board amendments from the University of Toronto and UNCST.
